# The acute effect of inhaled nitric oxide on the exercise capacity of patients with advanced interstitial lung disease: a randomized controlled trial

**DOI:** 10.1186/s12890-024-03051-4

**Published:** 2024-05-10

**Authors:** Lev Freidkin, Mordechai R Kramer, Dror Rosengarten, Shimon Izhakian, Shani Taieb, Barak Pertzov

**Affiliations:** 1 Pulmonary Division, Rabin Medical Center, Beilinson Campus, 39 Jabotinski St, Petach-Tikva, 4941492 Israel; 2 Internal medicine E, Rabin Medical Center, Petach Tikva, Israel; 3 Sackler Faculty of Medicine, Tel Aviv University, Tel Aviv, Israel

**Keywords:** ILD, Inhaled, Nitric Oxide, Pulmonary hypertension, Saturation, 6-minute walk test

## Abstract

**Background:**

Inhaled nitric oxide (iNO) selectively acts on the pulmonary vasculature of ventilated lung tissue by reducing pulmonary vascular resistance and intrapulmonary shunt. This effect may reduce ventilation/perfusion mismatch and decrease pulmonary hypertension in patients with interstitial lung disease.

**Methods:**

In a prospective, single-blinded, randomized, placebo-controlled trial, participants with advanced interstitial lung disease, underwent two separate six-minute walk tests (6MWT): one with iNO and the other with a placebo. The primary outcome measured the difference in meters between the distances covered in the two tests. Secondary outcomes included oxygen saturation levels, distance-saturation product, and Borg dyspnea score. A predefined subgroup analysis was conducted for patients with pulmonary hypertension.

**Results:**

Overall, 44 patients were included in the final analysis. The 6MWT distance was similar for iNO treatment and placebo, median 362 m (IQR 265-409) *vs* 371 m (IQR 250-407), respectively (*p* = 0.29). Subgroup analysis for patients with pulmonary hypertension showed no difference in 6MWT distance with iNO and placebo, median 339 (256-402) vs 332 (238-403) for the iNO and placebo tests respectively (*P*=0.50). No correlation was observed between mean pulmonary artery pressure values and the change in 6MWT distance with iNO versus placebo (spearman correlation Coefficient 0.24, *P*=0.33).

**Conclusion:**

In patients with advanced interstitial lung disease, both with and without concurrent pulmonary hypertension, the administration of inhaled nitric oxide failed to elicit beneficial effects on the six-minute walk distance and oxygen saturation. The use of inhaled NO was found to be safe and did not lead to any serious side effects.

**Trial registration:**

(NCT03873298, MOH_2018-04-24_002331).

**Supplementary Information:**

The online version contains supplementary material available at 10.1186/s12890-024-03051-4.

## Introduction

Nitric oxide (NO) is a molecule produced by the endothelial cells that modulates vascular tone by promoting the formation of cyclic guanosine 3',5'-monophosphate [1, 2]. When inhaled, nitric oxide gas (iNO) selectively acts on the pulmonary vasculature of ventilated lung tissue and rapidly binds to hemoglobin, resulting in no systemic effects. Unlike intravenous vasodilators, which can compromise hemodynamic stability, iNO reduces pulmonary vascular resistance and intrapulmonary shunt without affecting other organs [3–5]. Early randomized controlled trials (RCTs) in patients with pulmonary hypertension, including pediatric and adult populations [6–8], demonstrated the safety and efficacy of iNO and laid the foundation for subsequent studies exploring its benefits in diverse pulmonary, cardiac, and vascular conditions [9–12].

Interstitial lung disease (ILD) can lead to the development of pulmonary hypertension (PH) through hypoxic vasoconstriction and pulmonary vascular remodeling [13, 14]. As iNO can improve perfusion to ventilated lung areas and also reduces pulmonary vascular resistance it may be beneficial in the treatment of patients with ILD, and pulmonary hypertension associated with ILD (PH-ILD). A small trial that evaluated the effect of iNO on idiopathic pulmonary fibrosis (IPF) in seven patients found that inhaled nitric oxide reduced mean pulmonary arterial pressure during rest and exercise without disturbing gas exchange [15]. In 2020 Nathan et al published an RCT investigating iNO in patients with advanced pulmonary fibrosis at risk of pulmonary hypertension and found a positive effect in the iNO treatment group, which was more active in moderate to vigorous activity. However, with no beneficial effect in six minute walk test (6MWT) distance and oxygen saturation level [16]. In 2022 King et al published a small phase 2 randomized controlled trial with 44 patients with fibrotic ILD requiring oxygen and reported an increase in activity level, but similarly to Nathan et al. with no improvement in six-minute walk distance [17]. To date these are the only clinical trials published to evaluate iNO device in patients with ILD.

Given the limited evidence regarding iNO administration in ILD, this study aimed to assess the acute effect of inhaled NO on the exercise capacity and oxygen saturation of ambulatory patients with advanced ILD.

## Methods

We conducted a single-center, prospective, single-blinded, randomized, placebo-controlled trial at Rabin Medical Center in Israel between November 2019 and January 2023. The first patient was recruited on 03/11/2019, and the last patient was recruited on 15/01/2023. We included adult (age >18) patients with advanced ILD, that was defined as interstitial lung disease shown by high resolution chest CT and/or lung tissue biopsy that were diagnosed at a multidisciplinary discussion according to 2018 ATS/ERS/JRS/ALAT Clinical Practice Guideline [18]. Additional inclusion criteria were FVC below 80% and DLCO less than 60% of predicted in pulmonary function test performed at the day of study enrolment. Pulmonary function tests were conducted using the ZAN 300 nSpire Health pulmonary function testing system (Oberthulba, Germany). Lung volumes, including total lung capacity (TLC) and residual volume, were measured with a closed-type pressure plethysmograph. Diffusion capacity for carbon monoxide (DLCO) was assessed using the single-breath method with 0.3% carbon monoxide. all tests were performed in accordance with standard techniques and the guidelines of the American Thoracic Society/European Respiratory Society [19]. All measured parameters were expressed as a percentage of the predicted values according to the European Community for Coal and Steel [20]. Exclusion criteria were known moderate-severe systolic heart failure as defined by left ventricle ejection fraction of 40% and below, history of systemic sclerosis (scleroderma) and an inability to perform a 6-minute walk test. Patients' files were reviewed, demographic data and medical history including chronic medication, pulmonary function, echocardiographic, and right heart catheterization tests results were collected. All patients signed an informed consent form, and The research received approval from the Rabin Medical Center Institutional Review Board (RMC-0135-18) and the Ministry of Health (MOH_2018-04-24_002331). Additionally, it was registered in a clinical trial registry on 13/03/2019 (NCT03873298).

### Outcomes

The main outcome measure was the variation in distance covered during two six-minute walk tests performed by participants, one with inhaled nitric oxide (iNO) and one without. The initial primary outcome for this study was the change in oxygen saturation during the 6MWT (NCT03873298). However, given the challenges in adjusting the results for supplemental oxygen use and the fact that 6-minute walk test distance (6MWTD) was the primary outcome in key pulmonary hypertension (PHTN) trials [21–23], we have revised the primary outcome to be 6MWTD and we have designated SpO2 as a secondary outcome. This modification was implemented prior to patient recruitment. Secondary outcomes were the lowest saturation during the test, time of saturation below 90%, dyspnea score at the end of the test, adverse events, and the distance-saturation product (DSP). The DSP is calculated as a product of walked distance in meters and saturation nadir during 6 minute test [24]. A predetermined subgroup analysis was performed within the study population, focusing on patients with pulmonary hypertension. Pulmonary hypertension was defined as tricuspid regurgitation velocity (TRV) exceeding 2.8 m/s on transthoracic echocardiography (TTE) or mean pulmonary artery pressure (mPAP) surpassing 20 mm Hg measured through right heart catheterization (RHC) [25]. The study participants were blinded to the treatment used in each 6MWT. Study personal and data analysis were not blinded.

### Pulmonary hypertension measurement

The assessment of pulmonary hypertension was conducted using RHC or TTE. It is important to note that these examinations were not specifically performed for the purpose of this study but were retrieved from the patients' medical records. The patients underwent these tests as part of their evaluation for lung transplantation or as part of the ILD clinic evaluation. For the predefined subgroup analysis of patients with pulmonary hypertension, we included those with an mPAP greater than 20 mmHg as measured by RHC, and patients with a TRV exceeding 2.8 m/s as determined by TEE.

### Randomization and blinding

Upon obtaining informed consent from the patient, an opaque envelope was opened to determine whether the patient would undergo treatment with iNO in the first or second 6MWT. The patient was unaware of the treatment administered during each 6MWT. Study personnel were not blinded during the trial or the subsequent statistical analysis.

### Inhaled nitric oxide therapy

For delivery of inhaled NO a portable nitric oxide generator INOpulse (Bellerophon Therapeutics Inc. NJ, USA) was used. The INOpulse device administers pulsed doses of iNO through a specialized nasal cannula to subjects breathing spontaneously. It adjusts the pulse volume and frequency according to the subject's breathing rate, ensuring a consistent drug dosage per hour. This mechanism allows the device to deliver the precise μg/kg Ideal Body weight (IBW) dose hourly, regardless of variations in the subject's respiratory rate or tidal volume. The device is lightweight, easy to wear and allows to combine drug-delivery with oxygen external supply if needed. The active study drug, iNO, Nitric oxide for inhalation was supplied in size 0.074 liter aluminum cartridges at a concentration of 6.0 mg/L (4880 ppm). Placebo to match study drug was supplied in size 0.074 liter aluminum cartridges containing nitrogen (N2, 99.999%) gas. Both the nitric oxide and placebo cartridges were labeled to maintain blinding. Dose was administered according to Ideal Body weight (IBW), the first enrolled patient was treated at a dose of 45 mcg/IBW/h, for all other included patients the *iNOpulse* system was programmed for gas delivery at a dose of 75 mcg/kg-IBW per hour. For safety assessment, adverse events and serious adverse events were recorded. Continuous monitoring of pulse and SpO2 was conducted for each patient once connected to the iNO device. All patients were monitored during a 20-minute observation period before commencing the 6MWT, with the device administering either the drug or placebo during this time. This time frame was selected based on evidence indicating that a duration of 20 minutes is sufficient to facilitate the physiological effects of iNO [15]. The safety monitoring period was initiated from the onset of the observation period until 30 minutes following the cessation of drug delivery.

### Six-minute walk test

The study consisted of a single clinic visit during which each patient underwent two six-minute walk tests. One test was conducted with the iNO drug, and the other with a placebo. The tests were performed 30-60 minutes apart, and the order (whether the drug or placebo was given first in the 6MWT sequence) was determined through computer-generated randomization, with random numbers sealed in opaque envelopes. For the placebo 6MWT iNO generator was charged with a placebo cartridge, for the iNO 6-minute test - with nitric oxide cartridges. There was no visible difference between placebo and NO cartridges; both gases are odorless and colorless. The treatment with iNO and placebo started 30 minutes before the commencement of the 6MWT and ended at the end of the test. Patients that require supplemental oxygen performed the tests with oxygen supplementation.

The 6MWT was performed under investigators supervision and according to the official European Respiratory Society (ERS)/American Thoracic Society (ATS) guidelines [26, 27]. The pulse and saturation were recorded continuously from baseline to 5 minutes after the test ended. Adverse events, walk distance, and dyspnea severity were recorded as well. Dyspnea assessment was given by the patient according to the Borg score by scale from 0 (no dyspnea at all) to 10 (maximal exertion). Pulse and saturation were measured using Masimo Rad-57 portable pulse oximeter. To avoid movement artifacts, we used a forehead sensor with a headband to ensure proper skin contact with the probe. The measurement was continuous, and following the completion of the test, the data was transferred to a computer for thorough inspection for any inconsistencies in measurement and analysis.

### Statistical analysis

The necessary sample size for a paired analysis of the 6-minute walk test (6MWT) distance, with the clinically significant difference set at 30 meters, a standard deviation (SD) of 50 meters, and a significance level (alpha) of 0.05 with a power of 75%, was determined to be 44 patients [28]. Clinical and baseline characteristics are reported as frequency and percentage, means and SD or median and interquartile range (IQR). Clinical outcomes were compared with the paired samples student's t-test and the Wilcoxon signed rank test as appropriate. The correlation between 6MWT distance and severity of pulmonary hypertension was conducted with the spearman correlation test. *P* values of < 0.05 were considered significant. Statistical analysis was performed using SPSS software version 25.

## Results

Forty-five patients were enrolled in the study, one patient did not complete the second 6-minute walk test and was excluded. Therefore, 44 patients were included in the final analysis (Figure S1, supplementary data). Median age was 65.5 years (IQR 58-69), 31 patients (70%) were male. The underlying lung diseases were as follows: IPF in 20 patients (56%), idiopathic nonspecific interstitial pneumonia (iNSIP) in 5 patients (11%), combined pulmonary fibrosis and emphysema (CPFE) in 4 patients (9%), and sarcoidosis, cryptogenic organizing pneumonia (COP), hypersensitivity pneumonitis (HP), and connective tissue disease-associated interstitial lung disease (CTD-ILD) in 2 patients each (4.5%). Additionally, one patient each was diagnosed with graft-versus-host disease (GVHD) and unclassified ILD. The median FVC and DLCO were 57.5% (IQR 45-72%) and 38% (IQR 29-45%) of predicted respectively. Twenty-six patients were treated with antifibrotic therapy (59%), and twenty-three patients (52%) received anti-inflammatory treatment, 14 cases (31.8%) with prednisone, five cases (11%) with mycophenolate and 4 cases (9%) with rituximab. Eighteen (41%) patients required supplemental oxygen therapy. Pulmonary hypertension assessment was obtained using RHC and/or TTE in 36 patients. Among these, 22 patients underwent evaluation by RHC, revealing a median mPAP of 23 mmHg (IQR 19.25-27.75). Additionally, 17 patients had their systolic pulmonary artery pressure estimated through TRV via TTE, with a median TRV of 2.82 m/s (IQR 2.54-3.08). The baseline clinical and demographic characteristics are summarized in Tables 1 and 2.

**Table 1:** Baseline clinical and demographic characteristics

*N* = 44	**Median (IQR)**
Age, years	65.5 (58 – 69)
Male sex, n (%)	31 (70.5%)
FVC %predicted	57.5 (45 – 72)
DLCO %predicted	38 (29 – 45)
mPAP (RHC, *n* = 22) TRV (TTE, *n* = 17) sPAP (RHC and TTE, *n* = 38)	23 (19.25 – 27.75) 2.82 (2.54 – 3.08) 37 (30.00-47.25)
Time difference from study 6MWT to TTE, Months	6.7 (2.0-11.8)
Time difference from study 6MWT to RHC, Months	5.7 (4.4-17.7)
BMI, kg/m^2^	28 (25 – 30)
Supplemental oxygen therapy, n (%)	18 (41%)

*FVC* Forced vital capacity, *DLCO* Diffusing capacity for carbon monoxide, *mPAP* Mean pulmonary arterial pressure, *sPAP* Systolic pulmonary arterial pressure, *BMI* Body mass index, *TTE* Transthoracic echocardiography, *RHC* Right heart catheterization

**Table 2:** Underlying lung disease

	**n (%)**
**Underlying lung disease**	
IPF	25 (57)
iNSIP	5 (11)
CPFE	4 (9)
CTD-ILD	2 (4.5)
Sarcoidosis	2 (4.5)
COP	2 (4.5)
HP	2 (4.5)
GVHD	1 (2%)
Unclassifiable ILD	1 (2%)
**Drug therapy**	
Antifibrotic	
Nintedanib	23 (52)
Pirfenidone	3 (7)
Immunomodulatory	
OCS	14 (32)
MMF	5 (11)
Rituximab	4 (9)

*IPF* Idiopathic pulmonary fibrosis, *iNSIP* Idiopathic nonspecific interstitial pneumonia, *CPFE* Combined pulmonary fibrosis and emphysema, *COP* Cryptogenic organizing pneumonia, *HP* Hypersensitivity pneumonitis, *CTD-ILD* Connective tissue disease ILD, *GVHD* Graft *vs* host disease, *OCS* Oral corticosteroids, *MMF* Mophetil mycophenolate

**Fig. 1:**
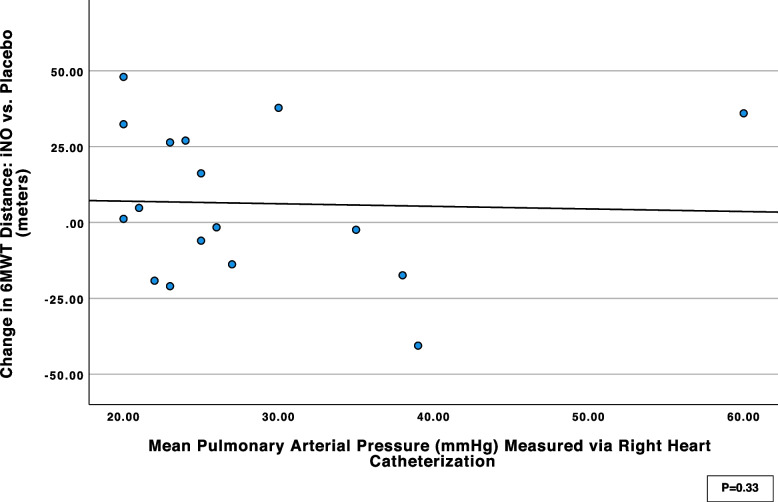
Correlation between mean pulmonary artery pressure and 6-minute walk distance, *n* = 22. Dot plot illustration of the mean pulmonary artery pressure of patients, as assessed through right heart catheterization, in relation to the change observed in their 6-minute walk distance, following the administration of inhaled nitric oxide (iNO) compared to a placebo. A positive value in the change of 6-minute walk distance indicates an improvement in the distance achieved with iNO compared to the placebo. Each dot on the plot represents an individual patient

The primary outcome, the 6MWT distance was similar for iNO treatment and placebo, median 362 m (IQR 275-410) *vs* 371 m (IQR 259-414), respectively (*p* = 0.29). The lowest oxygen saturation value during 6MWT was 86% (IQR 77-92) and 87% (IQR 78-94) with iNO and placebo respectively (*p* = 0.64). The median calculated DSP was 293 m% (IQR 232-378) and 290 m% (IQR 211-378) for iNO and placebo respectively (*p* = 0.6). The median BORG score was 3 (IQR 2-5) for both iNO and placebo (*p* = 0.58). The primary and secondary outcomes are presented in Table 3. Subgroup analysis for patients with pulmonary hypertension, defined as mPAP>20 mmHg by RHC and/or TRV>2.8 m/s by echocardiography, showed no difference in 6MWTD with iNO vs placebo, median 339 (256-402) vs 332 (238-403) for the iNO and placebo tests respectively (*n* = 26, *P*=0.50; Table 4). No correlation was observed between mPAP values and the change in 6MWT distance with iNO versus placebo (*n* = 22, spearman correlation Coefficient 0.24, *P*=0.33) (Fig. 1). Similarly, no correlation was found between systolic pulmonary artery pressure (sPAP) values, obtained from RHC and TTE, and changes in 6MWT distance with iNO versus placebo (*n* = 38, Spearman correlation coefficient: -0.10, *P*=0.55) (Figure S2, supplementary data).

**Table 3:** Primary and secondary outcomes

*N* = 44	**iNO, median (IQR)**	**Placebo, median (IQR)**	*P*value
6MWTD, m	362 (275-410)	371 (259-414)	0.29
DSP, m%	293 (232-378)	290 (211-378)	0.60
SpO_2_ nadir, %	86 (77-92)	87 (78-94)	0.64
Time SpO2 < 90%, minute	2.1 (0-4.6)	2.4 (0-4.4)	0.84
BORG	3 (2-5)	3 (2-5)	0.58

*6MWDT* Six-minute walk test distance, *DSP* distance-saturation product, *SpO*_*2*_ Oxygen saturation

**Table 4:** Subgroup analysis for patients with pulmonary hypertension

*N* = 26	**iNO, median (IQR)**	**Placebo, median (IQR)**	*P* value
6MWTD, m	339 (256-402)	332 (238-403)	0.50
DSP, m%	265 (199-323)	277 (200-349)	0.69
SpO_2_ nadir, %	86 (75-88)	87 (80-94)	0.84
Time SpO2 < 90%, minute	3.9 (0-4.68)	3.5 (0-4.45)	0.80
BORG	4 (3-5)	4 (3-6)	0.17

*6MWDT* Six-minute walk test distance, *DSP* distance-saturation product, *SpO*_*2*_ Oxygen saturation

The safety analysis set included all patients enrolled in the study, consisting of 44 patients who were included in the final analysis and one patient who was excluded for not completing two 6-minute walk tests. Overall, four patients experienced side effects during the study: three while under therapy with iNO and one while under placebo therapy. Specifically, one patient experienced sinus tachycardia with a heart rate exceeding 130 beats per minute during the iNO walk. Additionally, three patients reported dizziness during the 20-minute observation period prior to the initiation of the 6MWT. In one of these cases, the patient was on placebo therapy, and the dizziness resolved, allowing the completion of the 6MWT. In the cases involving iNO therapy, one patient's dizziness resolved, and they completed the 6MWT; in another case, iNO treatment was halted, the dizziness resolved within a few minutes, but the patient did not complete the 6MWT and was consequently excluded from the analysis. No episodes of symptomatic rebound or serious adverse events were observed. Overall, therapy with iNO at doses of 45 and 75 mcg/IBW/h was safe and well-tolerated.

## Discussion

In this prospective, single-blinded, randomized, placebo-controlled trial, treatment with iNO did not result in a significant difference in walk distance or oxygen saturation during 6MWT compared to placebo. Furthermore, no differences in outcomes were observed in a subgroup analysis of patients with pulmonary hypertension. These findings are consistent with those from two recent phase 2 RCTs that evaluated iNO therapy in patients with fibrotic interstitial lung disease (fILD). The first study, conducted by Nathan et al., showed that treatment with iNO at doses of 30, 45, and 75 μg/IBW/h resulted in no improvement in 6MWT distance, oxygen saturation, or DSP. However, it did note a significant improvement in moderate to vigorous activity (MVPA), an outcome that was not evaluated in this trial [16]. The second study, by King et al., involved patients with fILD who require oxygen supplementation and showed that iNO therapy at a dose of 45 μg/IBW/h did not improve 6MWT distance compared to placebo, but did result in improvements in physical activity levels and patient-reported outcomes [17]. The safety profile of inhaled nitric oxide (iNO) at doses of 45 and 75 mcg/IBW/h has been demonstrated to be favorable in the current study, with no serious side effects observed during the relatively brief treatment period of 30 minutes. This aligns with the findings from multiple previous trials that have also reported a positive safety profile for iNO therapy [3, 8, 15–17, 29–33]. Considering that the efficacy of iNO therapy in treating ILD and PH-ILD has not yet been conclusively established, this study underscores the feasibility of future trials aimed at exploring higher doses and extended treatment durations with iNO.

In this trial, the administration of iNO was relatively brief, with treatment initiation occurring 20 minutes prior to the 6-minute walk test. This contrasts with previously mentioned trials where patients underwent 16-24 hours of iNO treatment daily for several months [16, 17]. Our objective was to evaluate the immediate impact of iNO at a dose of 75 μg/IBW/h. Specifically, we aimed to determine whether iNO's ability to reduce pulmonary vascular resistance (PVR) as observed in previous studies in patients with IPF [15], translates into an immediate improvement in exercise tolerance. Our study revealed that iNO does not confer an immediate benefit, suggesting that the treatment may not be effective in ILD or that a longer duration of administration or a higher dose might be necessary to observe benefits. This lack of effect of iNO therapy was also observed in patients with primary pulmonary hypertension (PH) and PH secondary to cystic fibrosis [8, 33]. However, this is in contrast to chronic obstructive pulmonary disease (COPD), in which there is evidence suggesting that iNO increases 6MWTD and improves shortness of breath at rest and during exercise [30, 32].

Patients with ILD are at risk of developing pulmonary hypertension, which can lead to decreased exercise capacity, increased oxygen consumption, and worse outcomes [14]. Several trials have investigated whether drugs effective for primary PH (group 1) are also effective for PH-ILD (group 3), including Phosphodiesterase-5 (PDE_5_) inhibitors, Endothelin receptor antagonists (ERAs), soluble guanylate cyclase (sGC), and inhaled vasodilators. However, these drugs did not show significant results for PH-ILD [22, 34–37]. This lack of effectiveness for the above mentioned drugs and iNO may be explained by the severe parenchymal disease that limits the measurable effects of drug-induced reduced PVR. Treprostinil, the only treatment that displayed a positive impact on PH-ILD, further corroborate this theory, since it was shown to enhance the exercise capacity of patients with ILD without PH and reduce the rate of ILD exacerbations [23]. This implies that for a drug to truly benefit PH-ILD, it must exhibit activity on the underlying parenchymal lung disease and not just on the PH mechanism. Currently, the efficacy of Treprostinil in IPF is being assessed in a phase 3 trial [38]. Another potential reason for the ineffectiveness of iNO might be that traditional clinical trial outcomes, such as the 6MWT distance and oxygen saturation (SpO2), are insufficient to capture the therapeutic benefits of iNO in contrast to the innovative Actigraphy outcomes assessed in Nathan et al.'s study that demonstrated a positive effect and should be further explored and validated [16].

The trial has several limitations. First, the sample size was limited to only 44 patients with a power calculation of 75%. However, comparable in size to previous RCTs that evaluated the use of iNO in ILD [16, 17]. Second, the drug was evaluated for a short time period (20-30 minutes), which may not be adequate to produce a significant impact on the pathophysiology of the disease. Third, accurate measurement of pulmonary artery pressure with RHC was only conducted in 22 patients (47%), while transthoracic echocardiography was used for the remaining patients. Fourth, the outcomes used in this trial did not include actigraphy outcomes that showed significant benefit in previous trials with iNO [16]. Lastly, a learning effect for the 6-minute walk test has been previously described [27]. In our current study, we also observed this effect. An analysis of the 6-minute walk test distance, regardless of treatment allocation, showed that the distance walked in the second walk was significantly longer than the first walk (341 vs. 355 meters, *P* < 0.001). However, although this difference was statistically significant, it is below the minimal clinically meaningful difference estimated at approximately 28 meters [39]. Additionally, we believe that two factors have further mitigated the impact of this bias: first, the order in which iNO or placebo was used for the first and second 6MWTs was randomized; second, all patients had completed a 6MWT before enrollment in the trial as part of their previous clinical assessment.

## Conclusion

Inhaled nitric oxide demonstrated no significant effect on the exercise capacity of patients with ILD, nor were there differences in outcomes in patients with PH- ILD. The administration of iNO at a dose of 75 mcg/IBW/h was found to be safe, with no serious adverse effects observed, indicating the potential feasibility of higher doses or longer duration of therapy in future trials.

### Supplementary Information

Supplementary Material 1.

## Data Availability

The data that support the findings of this study are not openly available due to reasons of sensitivity and are available from the corresponding author upon reasonable request.
