# Supplementary material for: The acute effect of inhaled nitric oxide on the exercise capacity of patients with advanced interstitial lung disease: a randomized controlled trial
Source: BMC Pulm Med. 2024 May 10;24:226. doi: 10.1186/s12890-024-03051-4 (PMC11084010; doi:10.1186/s12890-024-03051-4)
Supplement: Supplementary file 1 — Supplementary Material 1. [file 12890_2024_3051_MOESM1_ESM.docx]

**Figure S1**

CONSORT (Consolidated Standards of Reporting Trials) diagram of patient disposition. iNO = 75 μg/kg ideal body weight/h.

**
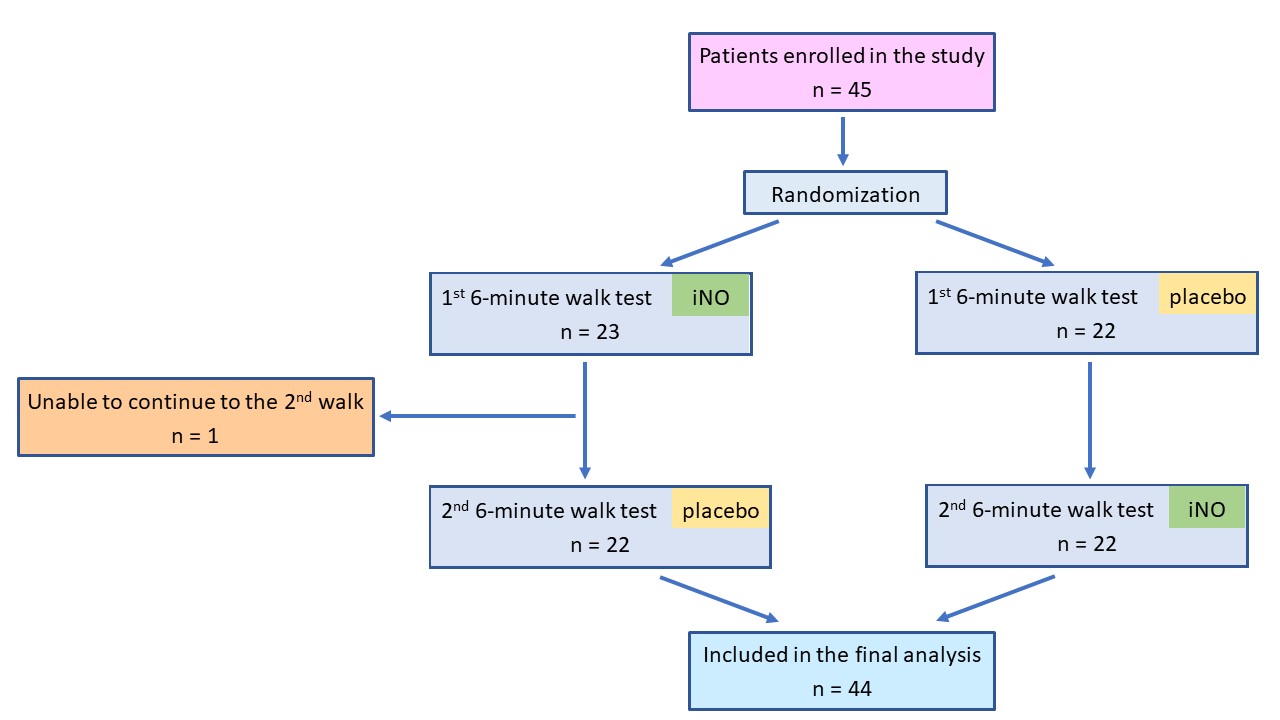
**

**Figure S2** Correlation between systolic pulmonary artery pressure and 6-minute walk distance, n = 36

Dot plot illustration of the systolic pulmonary artery pressure (sPAP, mmHg) of patients, as measured or estimated through right heart catheterization (RHC) or transthoracic echocardiogram (TTE) respectively, in relation to the change observed in their 6-minute walk distance (meter) following the administration of inhaled nitric oxide (iNO) compared to a placebo. A positive value in the change of 6-minute walk distance indicates an improvement in the distance achieved with iNO compared to the placebo. Each dot on the plot represents an individual patient.
